# Thermodynamic effect of single bubble near a rigid wall

**DOI:** 10.1016/j.ultsonch.2020.105396

**Published:** 2020-11-13

**Authors:** Qidong Yu, Xiaojian Ma, Zhicheng Xu, Jing Zhao, Dapeng Wang, Zhenwei Huang

**Affiliations:** a Department of Research and Development, China Academy of Launch Vehicle Technology, Beijing 100076, China; b State Key Laboratory of Hydroscience and Engineering, Department of Energy and Power Engineering, Tsinghua University, Beijing 100084, China

**Keywords:** Bubble oscillation, Thermodynamic effect, Temperature, High-speed jet, Counter jet

## Abstract

The objective of this paper is to numerically investigate the thermodynamic effect during bubble collapse near a rigid boundary. A compressible fluid model is introduced to accurately capture the transient process of bubble shapes and temperature, as well as corresponding pressure, and velocity. The accuracy of the numerical model is verified by the experimental data of bubble shapes, and Keller-Kolodner equation as well as its thermodynamic equation. The results show that a bubble near the rigid boundary presents high-speed jet in collapse stage and counter jet in rebound stage, respectively. In the collapse stage, the bubble margin will shrink rapidly and do the positive work on the compressible vapor inside the bubble, then a significant amount of heat will be generated, and finally the generation of high-speed jet drives the low-temperature liquid outside the bubble to occupy the position of high-temperature vapor inside the bubble. In the rebound stage, the counter jet moving away from the rigid boundary takes part of heat away from the sub-bubble, which avoids the external work of the expansion of the sub-bubble and the temperature reduction caused by the dissipation effect of the vortex structure. In addition, the initial standoff has a significant effect on the thermodynamics of bubble oscillation. The temperature keeps increasing with the increase of the initial standoff in the collapse stage, while it shows a downward trend with the increase of the initial standoff in the rebound stage. That’s because the high-speed jet and counter jet of bubble gradually disappear when the initial standoff increases, which is the important reason for the opposite evolution trend of temperature in collapse and rebound stage.

## Nomenclature

**Roman letters**
*a*: thermal diffusivity
*B*: fluid stiffness
*c*: sound velocity
*c_P_*: isobaric heat capacity
*c_V_*: constant heat capacity
*C*: phase function
*C_r_*: Courant number
***F****_TS_*: volume force
***g***: gravity acceleration
*Ga*: Gay-Lussac number
*h*: specific enthalpy
*H*: characteristic length
*k*: thermal conductivity
*L*: standoff distance
*Ma*: Mach number
*n*: calculated step
*P*: pressure
*Pe*: Peclet number
*P_0_*: initial pressure
*P_∞_*: pressure at infinity
*r*: radial distance from bubble center
*R*: instantaneous radius of a bubble
*R_m_*: initial radius of a bubble
*R_g_*: ideal gas constant
$\dot{R}$: first time derivative of *R*
$\ddot{R}$: second time derivative of *R*
*s*: specific entropy
*t*: time
*t_B_*: bubble period time
*t_OSC_*: Rayleigh oscillation time
*T*: temperature
*T_0_*: initial temperature
***u***: velocity vector
*u*: internal energy
*u_max_*: maximum horizontal velocity
***v***: vertical vector of velocity
*v*: specific volume
*v_max_*: maximum vertical velocity
*V_0_*: reference speed

**Greek letters**
*γ_g_*: ratio of specific heats
*δT*: reference temperature
*β*: thermal expansion coefficient
*λ*: thermal diffusivity
*μ*: viscosity coefficient
*μ_l_*: viscosity of the fluid
*μ_g_*: viscosity of the gas
*ρ*: density
*ρ_g_*: density of gas
*ρ_l_*: density of liquid
γ: normalized standoff distance
*χ_T_*: coefficient of adiabatic compression
*τ*: dimensionless time of bubble

**Special symbols**
*ΔP*: pressure difference
*Δt*: time interval of calculation
*Δx*: horizontal grid size
*Δy*: vertical grid size
*: normalized variable

## 1 Introduction

Cavitation is a typical phenomenon characterized by vapor generation and condensation occurrence in high-speed liquid flow, which seriously affects the operational efficiency and structural safety of hydraulic equipment, such as underwater weapons [1], aerospace device [2] and other fields [3], [4]. Thermodynamic effect of the cavitation is considered as one of most important reasons to cause the cavitation erosion and the structure damage [5] on the surface of the hydraulic equipment, which deserved to be deeply researched. Notably, cavitation phenomenon has multi-scale characteristics, which includes different forms, such as cavity shedding in convective scale and bubble pulsation in single bubble scale [6]. Compared to cavity shedding, the single bubble respectively performs in much smaller temporal scales (μs ~ ms) and spatial scales (μm ~ mm) [7], resulting in the technical difficulties and the obstacles of the thermodynamic measurements and studies.

Dular and Coutier [8] used a high-speed thermovision camera at 3 ~ 5 μm to measure the temperature variation in the liquid surrounding the single bubble in a cylinder partially filled with water at an ambient temperature and atmospheric pressure. The results showed that the temperature of the surrounding liquid decreases by 3 K in the process of cavitation growth, but increases by 4 K in the process of collapse. Flannigan and Suslick [9] carried out the investigations of high temperature ionization effect of bubble collapse by the sonoluminescence experiments, and they found that the emission temperature range caused by the bubble collapse is 4,000 ~ 15,000 K. Flint and Suslick [10] used spectroscopic probe to study the sonoluminescence spectra of ultrasonic cavitation bubble, and they found that the effective cavitation bubble temperature is 5075 ± 156 K. Liu et al. [11] studied the effect of liquid temperature on bubble collapse near the rigid boundary by focusing Q-switched laser pulse. The results showed that the impact pressure of liquid jet increases with the increase of liquid temperature, reaches the peak value, and then decreases. The increase is due to the change of physical properties of distilled water, and the decrease is due to the thermodynamic effect of bubble collapse and the change of mechanical properties of materials at high temperature. Generally, the different experimental principles of measuring equipment lead to obviously different experimental results of temperature, which brings significant technical resistance to reveal the mechanism of thermodynamic effect of single bubbles.

To further investigate the mechanism of single bubble thermodynamics, theoretical solution and analysis has been also conducted. The thermodynamic effect of single bubble is theoretically calculated based on the theory of Rayleigh-Plesset equation (R-P equation), which has been extensively applied to describe the bubble dynamics. For example, Brennen [12] investigated the temporal evolution of bubble temperature based on the R-P equation. Alhelfi and Sunden [13] used Keller-Kolodner equation and its thermal equation, a further extension of the R-P equation, to descript the temperature of single bubbles. Those theoretical models can just accurately describe the thermodynamic effect of spherical bubbles, but be invalid in simulating the non-spherical single bubbles near the rigid boundaries, which presents complex fluid structures, such as high-speed jet and counter jet [14]. Therefore, during actual research work of mechanism, those models just are used in verifying the accuracy of the numerical simulation method. In the recent years, significant progress in advanced flow simulation method has been made in understanding and modeling complex multiphase flows, especially for the thermodynamic effect of single bubble. Although Boundary Element Method (BEM) [15], Smooth Particle Hydrodynamics (SPH) [16], and Lattice Boltzmann Method (LBM) [17] have been applied in simulating bubble dynamics, the numerical simulation of investigating single bubble thermodynamics is a typical compressible problem, mainstreamedly realized by coupled solving the compressible Naiver-Stokes equations (N-S equation), phase equation, and interface capture model (like VOF and LS) [18]. Beig et al. [19] applied the compressible N-S equation to simulate a single bubble inertially collapsing near a rigid surface to measure the temperatures produced in the fluid. And they found that elevated temperatures along the wall can be produced by one of two factors, depending on the initial standoff distance of the bubble from the wall and the driving pressure. Kyriazis et al. [20] studied the thermodynamic effect of bubble collapse of n-dodecane by using the explicit density method of compressible Euler equation. The results show that the high-speed jet is produced in the later stage of n-dodecane bubble collapse, and the temperature is as high as 1000 K. Qin et al. [21] established the compressible N-S equation of single bubble collapse, and studied the transient changes of gas fraction, temperature, pressure and velocity distribution in the bubble and liquid. In addition, by modifying the R-P equation, they calculated quantitatively the temperature change in the process of single cavity rupture. Christian et al. [22] used compressible simulation method to study the transient evolution process of laser-induced cavitation, and assumed that all the vapor in the bubble is ideal gas. However, this model leads to overestimation of the size of the bubble rebound stage in the simulation of bubble collapse, so those models are limited to simulations of isentropic process.

In present work, based on the compressibility of gas-iquid two-phase fluids induced by bubble collapse, the multi-order partial differential equation of pressure with respect to velocity, temperature and density is derived, and then the conservation equations of mass, momentum and energy in the calculation domain are rebuilt, in order to provide reference data for the study of thermodynamic effect in the process of cavitation collapse. The purpose of this paper is (1) to study the compressible model to accurately simulate the thermodynamic effect of single bubble oscillation and introduce the verification method based on the Keller-Kolodner equation and its thermal equation; (2) to explore the temporal evolution of the temperature field in the stage of bubble collapse and rebound in detail; (3) to compare the effect of different initial positions on the thermodynamic effect of bubble.

## 2 Numerical methodology

### 2.1 Governing equation

According to the compressible characteristics of bubble and the weakly compressible characteristics of liquid, we used an updated numerical model to the field of simulate the bubbles and its thermodynamics. In this paper, the updated multiphase model proposed by Caltagirone et al. [23] was used to simultaneously calculate the weakly compressible liquid motion and strongly compressible gas motion with one set of N-S equation. As the gas flow inside the bubble is compressible, a compressible model has to be built to describe at the same time the vapor and the two-phase character of the problem. A mixed model capable of simultaneously managing compressible gas motions and the weakly compressible liquid features of the two-phase flow is required, which can save a lot of computing resources. And this method has been used in our previous work, which can be reviewed in Ref. [24]. In this part, Eqs. (1), (2), (3), (4), (5), (6), (7), (8), (9), (10), (11), (12), (13), (14), (15) are main framework of the algorithm model derived by Caltagirone et al. [23] and some necessary steps are further derived in this work. We would briefly introduce the derivation process in order to the convenient reading for readers.

#### 2.1.1 Mass equation

The fluid medium in the whole flow field is regarded as a mixture of water and gas with density variable. The ideal equation of state is

(1) $$P+\gamma_{g}B=\rho R_{g}T$$

where *P* is the pressure, *γ_g_* is the ratio of specific heats, *B* is the fluid stiffness, *ρ* is the density, *R_g_* is the ideal gas constant, and *T* is the temperature. Therefore, Eq. (1) can be expressed as the derivative form of temperature and density function to time

(2) $$\begin{array}{c} \frac{\mathrm{dP}}{\mathrm{dt}}={\left(\frac{\partial P}{\partial T}\right|}_{\rho }\frac{\mathrm{dT}}{\mathrm{dt}}+{\left(\frac{\partial P}{\partial \rho }\right|}_{T}\frac{d\rho }{\mathrm{dt}}\\ \frac{\mathrm{dP}}{\mathrm{dt}}=\frac{\beta }{\chi_{T}}\frac{\mathrm{dT}}{\mathrm{dt}}+{\left(\frac{\partial P}{\partial \rho }\right|}_{T}\frac{d\rho }{\mathrm{dt}} \end{array}$$

where *t* is the time; $\beta \;\text{=}\;\frac{1}{v}{\left(\frac{\partial v}{\partial T}\right)}_{P}$ is the coefficient of thermal expansion where *v* is specific volume; *χ_T_* is the coefficient of adiabatic compression with definition of $\frac{1}{\chi_{T}}\;\text{=}\;\rho {\left(\frac{\partial P}{\partial \rho }\right|}_{T}$. And the compressible mass conservation equation is defined as

(3) $$\frac{d\rho }{\mathrm{dt}}+\rho \nabla \cdot u=0$$

where ***u*** is the velocity vector. Substituting Eq. (3) into Eq. (2)

(4) $$\frac{\mathrm{dP}}{\mathrm{dt}}=\frac{\beta }{\chi_{T}}\frac{\mathrm{dT}}{\mathrm{dt}}+{\left(\frac{\partial P}{\partial \rho }\right|}_{T}\left(-\rho \nabla \cdot u\right)$$

where

(5) $$\frac{\mathrm{dP}}{\mathrm{dt}}=\frac{\partial P}{\partial t}+u\cdot \nabla P$$

So, Eq. (4) can be expressed as

(6) $$\frac{\partial P}{\partial t}=\frac{\beta }{\chi_{T}}\frac{\mathrm{dT}}{\mathrm{dt}}-\frac{1}{\chi_{T}}\nabla \cdot u-u\cdot \nabla P$$

After a series of strict formula derivation in Appendix A, Eq. (6) can be simplified into

(7) $$\frac{\partial P}{\partial t}=-\frac{1}{\chi_{T}}\nabla \cdot u$$

The Eq. (7) is the updated mass equation, which is derived based on the mass conservation and equation of state.

In their work, Vincent et al. [25] further qualitatively explained the operation mechanism of the updated mass equation in the aspect of simultaneously managing strongly and weakly compressible fluid motions. The detailed description is as follows: concerning the mass conservation of Eq. (10), it can be noticed that, in the condition of incompressibility of liquid, *χ_T_* is small and hence 1/*χ_T_* is large enough to render ∂p/∂t negligible. Therefore, the mass conservation equation is almost treated as incompressible. In the condition of strong compressibility of gas, *χ_T_* is significantly larger and mass conservation equation becomes the standard compressible pressure equation in which the isobaric dilatation term is discarded due to its negligible magnitude. Actually, the value of *χ_T_* of liquid around the bubble is neither extremely infinite nor infinitesimal, resulting the Eq. (7) performing the weak compressibility investigated in our work [26].

#### 2.1.2 Momentum equation

The momentum equation of the incompressible fluid model can be expressed as follows

(8) $$\rho \left(\frac{\partial u}{\partial t}+\left(u\cdot \nabla \right)u\right)=-\nabla P+\rho g+\nabla \left(\mu \left(\nabla u+\nabla^{T}u\right)\right)+F_{\mathrm{TS}}$$

where *μ* is the viscosity coefficient, ***g*** is the gravity acceleration, and ***F****_TS_* is the volume force. In the process of solving the above equation, the pressure Poisson equation needs to be introduced to solve, which significantly increases the calculation amount and reduces the calculation efficiency. To solve the above problems, substituting Eq. (7) into Eq. (8) can obtain

(9) $$\rho \left(\frac{\partial u}{\partial t}+\left(u\cdot \nabla \right)u\right)=-\nabla \left(P-\frac{\Delta t}{\chi_{T}}\nabla \cdot u\right)\;\text{+}\;\rho g+\nabla \left(\mu \left(\nabla u+\nabla^{T}u\right)\right)+F_{\mathrm{TS}}$$

where *Δt* is the characteristic length of the calculation time.

#### 2.1.3 Energy equation

The incompressible energy equation can be expressed as

(10) $$\rho c_{P}\left(\frac{\partial T}{\partial t}+u\cdot \nabla T\right)=\nabla \cdot \left(\lambda \nabla T\right)+\beta T\frac{\mathrm{dP}}{\mathrm{dt}}$$

where *c_P_* is the isobaric heat capacity, and$\lambda \;\text{=}\;\frac{k}{\rho c_{v}}$ is the thermal diffusivity where *k* is the thermal conductivity and *c_V_* is the constant heat capacity. The second term on the right side of the equal sign should be expressed in the form of expansion as shown in Eq. (4),

(11) $$\beta T\frac{\mathrm{dP}}{\mathrm{dt}}=\beta T{\left(\frac{\partial P}{\partial T}\right|}_{\rho }\frac{\mathrm{dT}}{\mathrm{dt}}+\beta T{\left(\frac{\partial P}{\partial \rho }\right|}_{T}\frac{d\rho }{\mathrm{dt}}$$

The first item of the right hand of the equation can be expressed as

(12) $$\beta T{\left(\frac{\partial P}{\partial T}\right|}_{\rho }=\rho \left(c_{P}-c_{V}\right)$$

The detailed derivation process of Eq. (12) can be found in Appendix B. Combining Eq. (4) and Eq. (12), Eq. (11) can be expressed as

(13) $$\beta T\frac{\mathrm{dP}}{\mathrm{dt}}=\rho \left(c_{P}-c_{V}\right)\left(\frac{\partial T}{\partial t}+u\nabla \cdot T\right)-\frac{\beta T}{\chi_{T}}\nabla \cdot u$$

Therefore, the form of compressible energy equation can be expressed as

(14) $$\rho c_{V}\left(\frac{\partial T}{\partial t}+u\cdot \nabla T\right)=\nabla \cdot \left(\lambda \nabla T\right)+\frac{\beta T}{\chi_{T}}\nabla \cdot u$$

#### 2.1.4 Governing equations of multiphase flow

The governing equations discussed above are all the single fluid models, which just can solve the dynamic characteristics of the single fluid. However, the bubble behaviors involve two different kinds of fluids, namely, gas and liquid. Therefore, in order to obtain the information of gas and liquid at same time, phase function should be used to solve the two-phase flow in the framework of single fluid N–S equations.

**Fig. 1:**
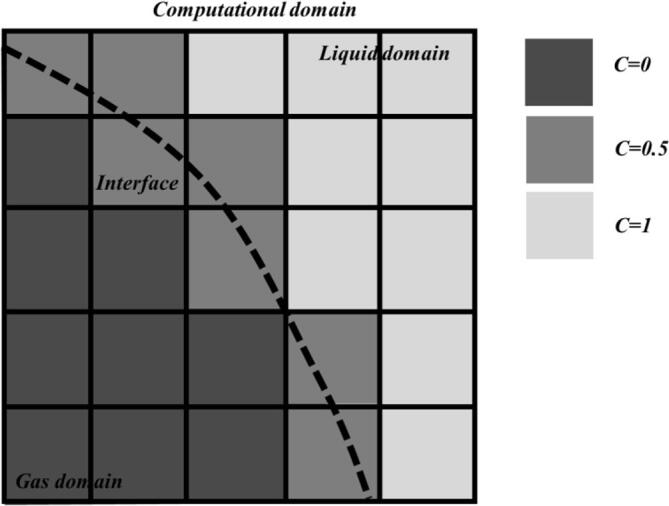
Schematic drawing of multiphase flow domain with phase function C.

As shown in Fig. 1, the N–S equations of solving multiphase flows are used in the whole computational domain with three fictitious domains, namely, gas, liquid, and interface domains. An equivalent fluid containing all the phases, i.e. gas domain and liquid domain, is built thanks to a phase function which depends on time and space. Therefore, the governing equations of multiphase flow can be expressed as

(15) $$\left\{\begin{array}{c} \frac{\partial P}{\partial t}+\frac{1}{\chi_{T}}\nabla \cdot u=0\\ \tilde{\rho }\left(\frac{\partial u}{\partial t}+\left(u\cdot \nabla \right)u\right)=\tilde{\rho }g-\nabla \left(P-\frac{\tau_{c}}{\chi_{T}}\nabla \cdot u\right)+\nabla \left(\tilde{\mu }\left(\nabla u+\nabla^{T}u\right)\right)+F_{\mathrm{TS}}\\ \tilde{\rho }c_{v}\left(\frac{\partial T}{\partial t}+u\cdot \nabla T\right)=\nabla \cdot \left(\tilde{\lambda }\nabla T\right)+\frac{\beta T}{\chi_{T}}\nabla \cdot u\\ \frac{\partial C}{\partial t}+u\cdot \nabla C=0 \end{array}\right)$$

where the density, viscosity and conductivity of the mixed multiphase flow are defined as $\tilde{\rho }=C\rho_{l}+\left(1-C\right)\rho_{g}$,$\tilde{\mu }=Cu_{l}+\left(1-C\right)u_{g}$, and $\tilde{\lambda }=C\lambda_{l}+\left(1-C\right)\lambda_{g}$, and the subscripts *l* and *g* represent the liquid phase and gas phase respectively. Phase function *C* is 1 for liquid phase and 0 for gas phase. The interface between liquid phase and gas phase is defined as *C* = 0.5. The VOF method was used as a multiphase flow model to track the gas–liquid interface, in order to keep the interface between the two phases clear when the topological structure of the interface changes, so as to obtain the flow details of the fluid field [27].

### 2.2 Model establishment and initial condition setting

**Fig. 2:**
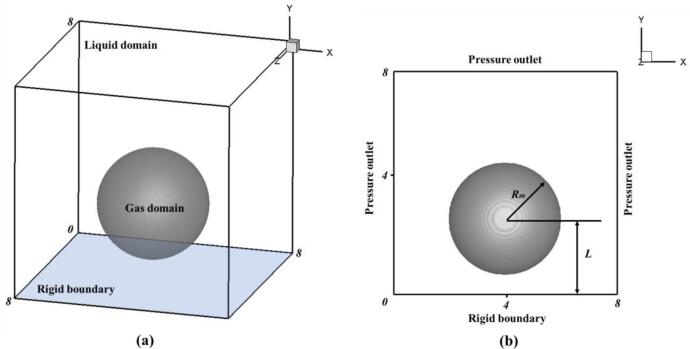
Schematic drawings of geometric model: (a) 3D area division of multiphase flow; (b) 2D settings of boundary conditions and initial conditions.

As shown in Fig. 2, the physical problems studied in this paper can be summarized as the oscillation process of a single spherical bubble near the horizontal rigid bottom driven by the internal and external pressure difference. In this paper, the compressible gas–liquid two-phase flow method is used to simulate the oscillation process of bubble near the rigid boundary. It is assumed that there is no condensable gas inside the bubble, but only compressible vapor which can be regarded as ideal gas. Liquids are considered to be compressible and completely static. Considering the viscosity of liquid and vapor, the whole flow process is considered as laminar flow. And the mass exchange process is not considered between vapor and flow field inside the bubble. In order to simplify the representation of the bubble size and the distance to the wall, a normalized standoff distance is used

(16) $$\gamma =\frac{L}{R_{m}}$$

where *R_m_* is the initial radius of the bubble, and *L* is the distance between the bubble center and the fixed wall at the initial time. The lower boundary of the calculation domain is defined as the rigid wall boundary, and the slip free boundary condition is adopted; the other five boundary surfaces are all set as pressure outlets.

**Fig. 3:**
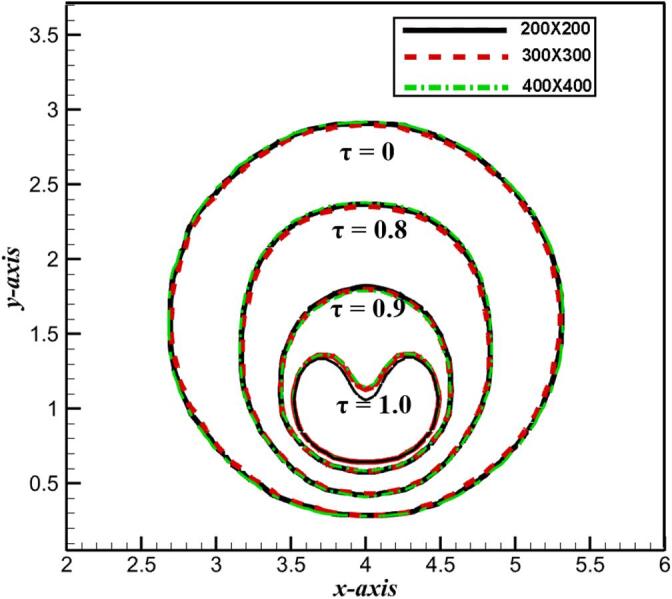
Mesh size independence verification of bubble shapes near a wall at typical times: τ = 0, 0.8, 0.9 and 1.0 (γ = 1.2).

In order to simulate a more accurate collapsing bubble shape near a rigid wall, especially the dynamics of high-speed jet, the independence of mesh sizes is verified in this section. Actually, mesh size was verified in our previous work [24]. In order to further obtain the accurate results, much tighter grids are adopted and investigated on the effect of bubble shapes, such as 200 × 200, 300 × 300 and 400 × 400, respectively. The normalized standoff distance is γ = 1.2. Meantime, a normalized collapse time of bubble *τ* is defined [28],

(17) $$\tau =\frac{t_{B}}{t_{\mathrm{OSC}}}$$

where *t_B_* is the bubble period time, and *t_OSC_* is defined as Rayleigh oscillation time. The typical bubble profiles are extracted at four time of *τ* = 0, 0.8, 0.9 and 1.0 under various mesh size resolutions. Fig. 3 shows the temporal evolution of the bubble shape from the initial state to the collapse at different mesh resolutions. It can be found that the collapsing bubble shapes near a wall almost coincides with each other under different mesh sizes, except for the slight difference of high-speed jet tip when 200 × 200 and *τ* = 1.0. Considering the efficiency and accuracy of numerical simulation, the numerical simulation is carried out in mesh size of 300 × 300 in this paper.

The time interval *Δt* at each calculated step used in present work is not a constant, but will change along the physical properties of simulation process. The time interval at each calculation instant is adapted by two restrictions to ensure stable and convergent numerical results, i.e. physical characteristics (the maximum velocity and density) and the calculation gird sizes, which cannot travel a distance more than one cell size within one-time step. Therefore, the time steps can be rationalized as long as the grid sizes are appropriate. Then, the time step reported by Nichols et al. [29] was used in present work,

(18) $$\Delta t=\min\left[C_{r}\frac{\Delta x}{\left|u_{\max}\right|},C_{r}\frac{\Delta y}{\left|v_{\max}\right|},\sqrt{\min\left(\rho_{l},\rho_{g}\right)\cdot \min{\left(\Delta x,\Delta y\right)}^{3}}\right]$$

where *C_r_*, the Courant number, was set to be 0.25 in our calculations; *u_max_* and *v_max_* are the maximum values of the horizontal and vertical velocities at each calculation instant, respectively. *Δx* and *Δy* are the grid sizes in horizontal and vertical directions, respectively.

### 2.3 Verification of calculation model

**Fig. 4:**
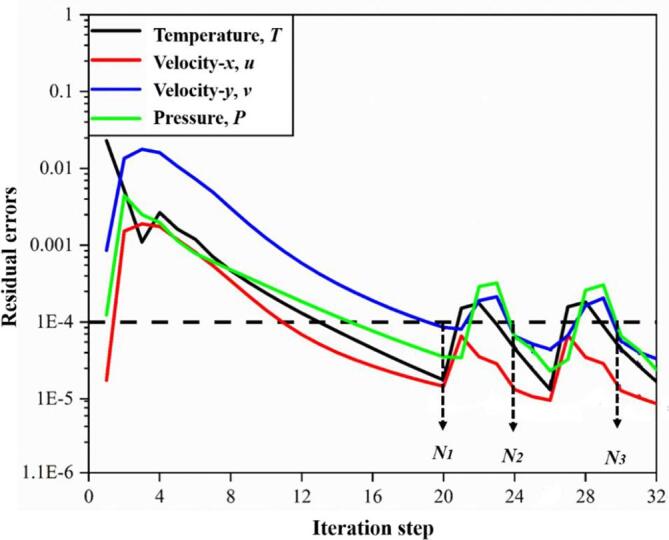
Variations of residual errors along iteration steps in pressure, velocity and temperature fields.

In order to verify the stability and reliability of the compressible numerical method, the flow field residual convergence of bubble oscillation in the free field is given. Fig. 4 shows the convergence curve of the residual of the first three timesteps. In this paper, the convergence target of temperature, velocity and pressure is set to 10^-4^. After about 20 iterations, the temperature, velocity and pressure residuals of the numerical model are reduced to 10^-4^. At this time, the flow field information of *N_1_* time step is calculated and converged. Taking the flow field information obtained in *N_1_* time step as the initial value, the iteration calculation of next time step *N_2_* is then carried out. The flow field information at the next time point is calculated by analogy until the set time step is reached. Therefore, it is considered that the method used in this paper has good convergence when calculating the stability of numerical solution of nonlinear problems, and its calculation results are stable and reliable.

In order to further prove the accuracy of methodology in simulating the bubble thermodynamics, the Keller-Kolodner equation is used to verify the compressible bubble model mentioned above. For more details on the derivation of bubble radius equation can be seen as [13],

(19) $$R\ddot{R}\left(1-\frac{\dot{R}}{c}\right)+\frac{3}{2}{\dot{R}}^{2}\left(1-\frac{\dot{R}}{3c}\right)=\left(1+\frac{\dot{R}}{c}\right)h+\frac{R}{c}\frac{\mathrm{dh}}{\mathrm{dt}}$$

In the formula, *R* is the instantaneous radius of the bubble, $\dot{R}\;\text{=}\;\frac{\text{d}R}{dt}$ is the velocity of bubble margin; $\ddot{R}\;\text{=}\;\frac{{\text{d}}^{2}R}{dt^{2}}$ is the acceleration of bubble margin, and *h* is the enthalpy of the liquid. And the energy equation is required, which is defined as following [13]

(20) $$\frac{\partial T}{\partial t}+\frac{R^{2}\dot{R}}{r^{2}}\frac{\partial T}{\partial r}=\frac{\lambda }{r^{2}}\frac{\partial }{\partial r}\left(r^{2}\frac{\partial T}{\partial r}\right)$$

where *r* is the radial distance from the center of the bubble.

**Fig. 5:**
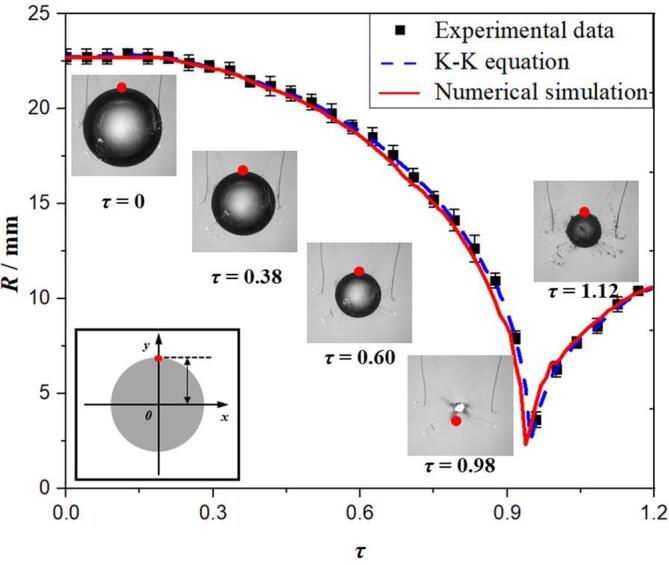
Comparions of experimental, theoretical and numerical results of spherical bubble radius in free field.

**Fig. 6:**
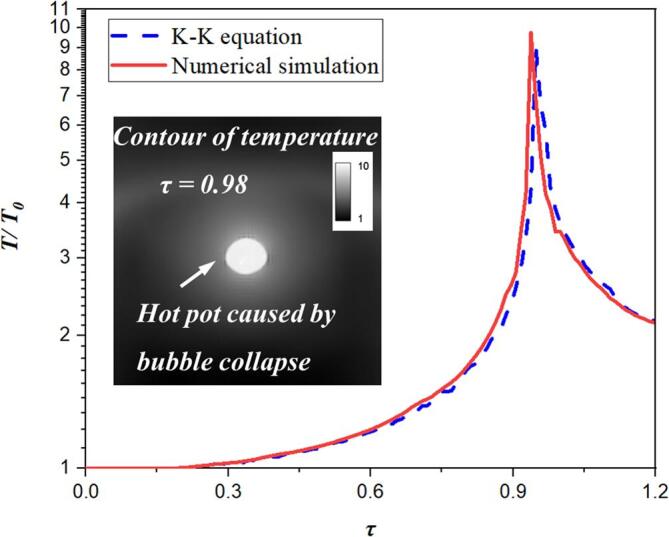
Comparisons of theoretical and numerical results of collapsing temperature at the bubble center.

Fig. 5 shows the experimental, numerical and theoretical comparison of the bubble radius in the free field with the same initial parameters. Among them, the maximum radius is *R_m_* = 23 mm, *P_∞_* = *P_l_* = 0.1013 MPa, and other parameters are shown in Table 1. Set the lower wall boundary in Fig. 2 as the pressure outlet. In order to verify our numerical model is accurate, two steps are performed, including verifications of bubble shapes and thermodynamics, respectively. For the former shown in the Fig. 5, the numerical solution of the bubble radius is much consistent with the experimental and theoretical solution, which could verify the accuracy of the numerical simulation method established in this paper. It notes that the experimental images of bubble shapes used in present work are all obtained by electric spark [30], which can easily ensure the consistency with the numerically initial settings with parameters *γ*, *P*, and ***u***. In order to further verify the latter one, Fig. 6 shows the numerical and theoretical comparison diagram of thermodynamic effect of bubble oscillation in the free field. The temperature trends between two methods are almost in good agreement, which shows the numerical simulation method mentioned above is correct for the temperature simulation of bubble collapse.

**Table 1:** Initial parameter settings.

Parameters	Symbol	Unit	Value
Pressure in liquid	*P_l0_*	MPa	0.1013
Pressure in gas	*P_g0_*	MPa	0.0031
Temperature in liquid	*T_l0_*	K	300
Temperature in gas	*T_g0_*	K	300
Density in liquid	*ρ_l_*	kg/m^3^	980
Density in gas	*ρ_g_*	kg/m^3^	1
Sound speed	*c*	m/s	1500

## 3 Results and discussions

### 3.1 Typical bubble shapes and flow structures

**Fig. 7:**
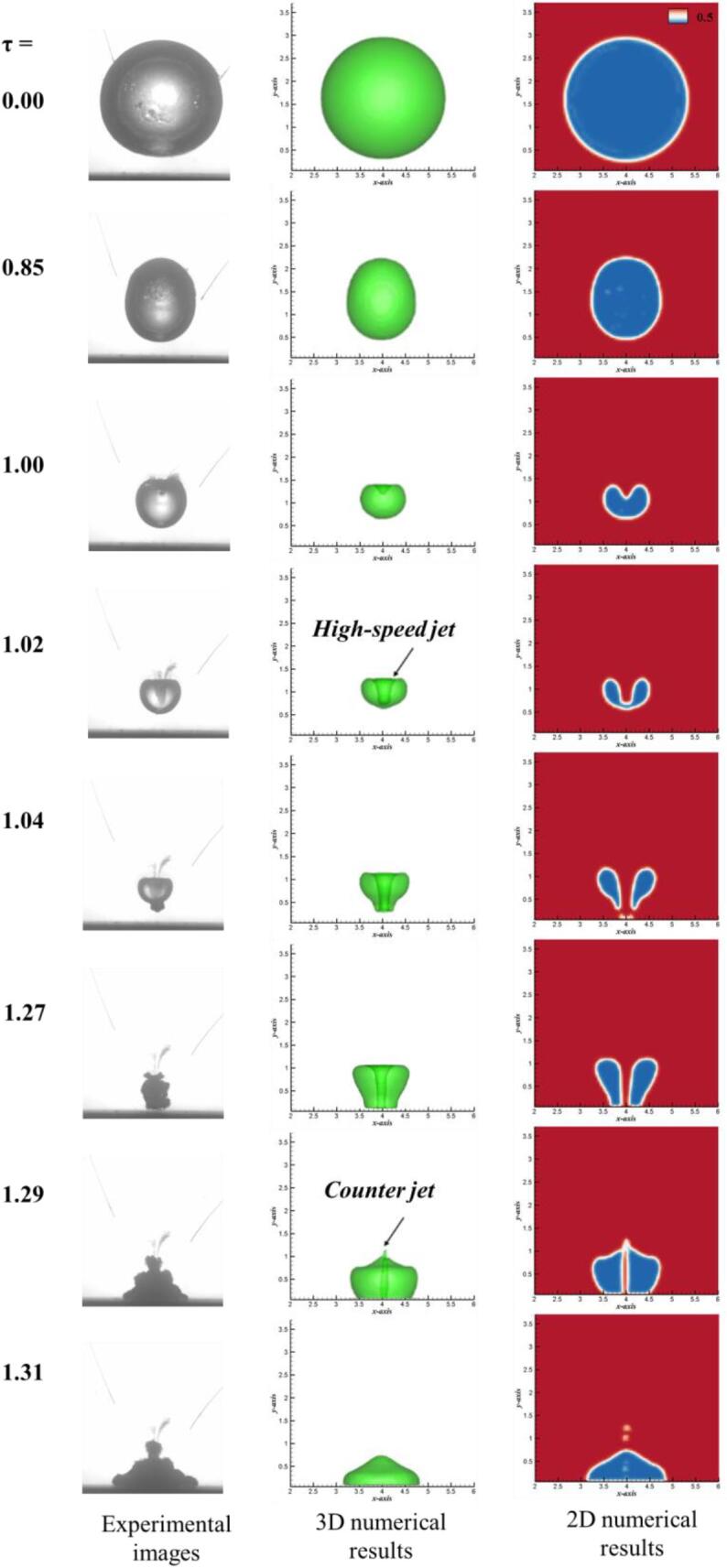
Comparisons of experimental bubble shapes (left), 3D numerical gas-liquid interface (middle) and 2D gas-liquid interface (right) (γ = 1.2).

Fig. 7 shows the comparison of temporal bubble shapes between the experimental results and the numerical prediction results when the initial position is γ = 1.2. In the process of numerical simulation, the initial condition is that the distance between the center of the bubble and the fixed wall is *L* = 21.36 mm and the initial radius of the bubble is *R_m_* = 17.80 mm. In addition, the initial temperature, density, pressure, sound velocity and other parameters in the gas–liquid phase are shown in Table 1. As shown in Fig. 7, the first column is the experimental data, the second column is the 3D gas–liquid interface, and the third column is the 2D gas–liquid interface of the numerical simulation results. It notes that the 2D gas–liquid interface is a cross-section extracted from center of 3D results. According to the variations of the typical bubble shapes, the bubble oscillation is divided into two typical stages: collapse and rebound. In the collapse stage, the volume of the bubble mainly performs contraction and high-speed jet. For example, when *τ* = 0.00, the initial shape of the cavity near the rigid boundary is spherical. When *τ* = 0.85, the lower margin of the bubble almost adheres to the rigid boundary, and the rest of the bubble surface shrinks rapidly. Especially for the left and right edge of the bubble, the contraction speed is faster than the top margin of the bubble, which leads to the ellipsoidal contraction of the bubble. When τ = 1.00, the lower surface of the bubble presents a flat shape, while the upper surface of the cavity has a larger collapse speed, resulting in the continuous downward movement of the upper surface of the bubble. When τ = 1.02, the top margin of the bubble strikes the lower margin, forming a high-speed jet. When τ = 1.04, in the final stage of bubble collapse, the high-speed jet penetrates the lower surface of the bubble. For the rebound stage, when τ = 1.27, the lower end of the jet of the bubble impacts on the rigid boundary, and the bubble still remains annular shape. When τ = 1.29, the top of the bubble produces a counter jet away from the rigid wall, and the volume of the bubble expanded for a second time. When τ = 1.31, the counter jet keeps moving upwards and the sub-bubble stays a pyramid shape. The numerical prediction of the bubble body has a good agreement with the experimental measurement results, except for the slight difference of sub-bubble caused by the rebound when τ = 1.29. The difference between the experimental and numerical results mainly results from the disability of front-tracking method when the gas–liquid interface changes from simply connected domain to multiply connected domain, especially during the rebound stage of the bubble. Compared with simply connected domain, multiply connected domain can take a worse numerical algorithm to construct the gas–liquid interface of volume fraction, which cannot ensure the good conservation of interface results.

**Fig. 8:**
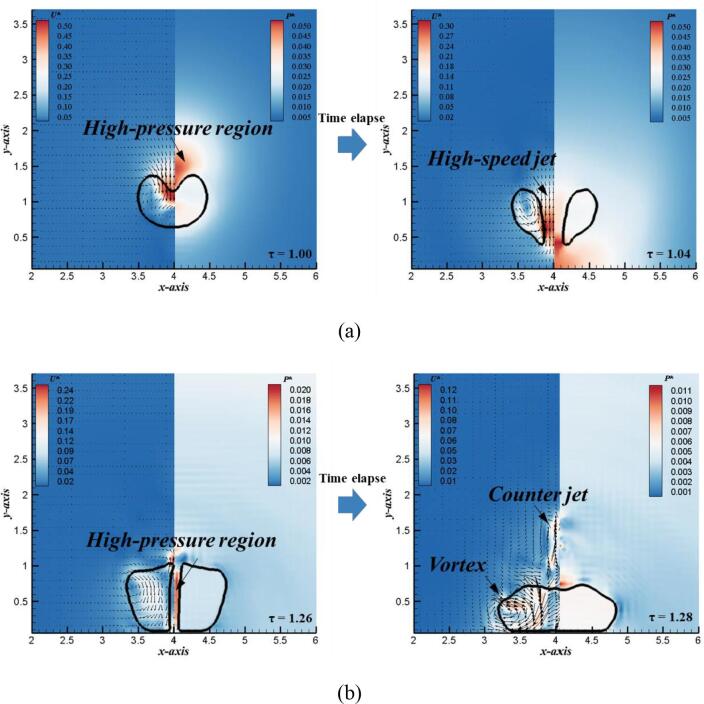
Numerical velocity (left) and pressure (right) distributions of bubble at typical times to illustrate (a) the formation of high-speed jet, and (b) the formation of counter jet (γ = 1.2).

In order to further explain the generation mechanism of the typical flow structures of single bubble in different stages, Fig. 8 shows the bubble morphology, pressure and velocity vector diagram of collapse and rebound stages, respectively. As shown in Fig. 8 (a), when the single bubble is in the collapse stage, a high-pressure region appears at the top of the bubble margin, which is the important reason to generation of the high-speed jet. The high-pressure region pushes the high-speed jet to puncture the bottom surface of the bubble. The velocity vector diagram shows that the high-speed jet impinges on the rigid wall, while the vapor inside the bubble forms a typical vortex motion inside the annular bubble. The generation of high-speed jet drives the liquid outside the bubble to occupy the position of the gas inside the bubble. Fig. 8 (b) further shows the bubble shape, pressure and velocity vector during the rebound stage. At this stage, there are two typical flow structures in the bubble: one is the counter jet moving away from the rigid wall at the top of the bubble, and the other is the vortex structure around the sub-bubble caused by interaction of fluid flow and rigid wall. The formation of the counter jet is due to the high-pressure region generated inside the annular bubble, which pushes the liquid inside to move upward. Furthermore, under the restrictions of the rigid wall, the circulation flow inside the bubble moves around along the wall shown by velocity vectors, resulting in the formation of the vortex structure.

### 3.2 Thermodynamic effect of bubble oscillation

**Fig. 9:**
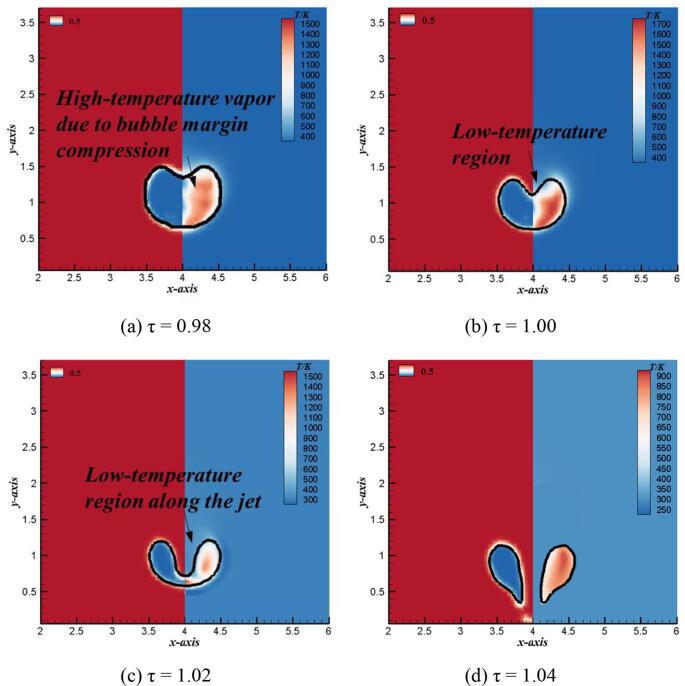
Transient evolutions of numerical bubble shapes (left) and temperature distributions (right) during bubble collapse stage (γ = 1.2).

In this part, the thermodynamic characteristics of bubble in collapse and rebound stages will be introduced in details, respectively. Fig. 9 shows the comparison of numerical temperature and bubble shapes in the collapse stage. As shown in Fig. 9 (a), due to the rapid reduction of the bubble volume in the collapse stage, the bubble margin pushes the compressible vapor inside the bubble and does positive work on it. Because the bubble collapse stage is very short, usually in milliseconds or even microseconds [16], the heat cannot be transferred to the low-temperature fluid outside the bubble by passing through the margin of the bubble in time, resulting in the rapid temperature rise of the compressible vapor inside the bubble. As shown in Fig. 9 (b), the top margin of the bubble is depressed downward, forming a high-speed jet. The low temperature liquid in the flow field occupies the high temperature area of the bubble along with the movement of high-speed jet, and the temperature in this area decreases rapidly. Fig. 9 (c) shows that when the top of the high-speed jet almost impinges on the bottom surface of the bubble, the low-temperature fluid outside the bubble further occupies the high-temperature region inside the bubble. At the same time, the bubble almost shrinks to the minimum volume, and the margin of the bubble continuously does works on the compressible vapor inside it, so the temperature in the bubble reaches the a very large temperature. Fig. 9 (d) shows that the vapor with high temperature inside the bubble is stored in the annular bubble when the top of the high-speed jet punctures the bottom margin of the bubble. At this time, the heat exchange between the high temperature vapor in the bubble and the low temperature liquid outside the bubble is obvious, especially the temperature at the region of high-speed jet is significantly improved.

**Fig. 10:**
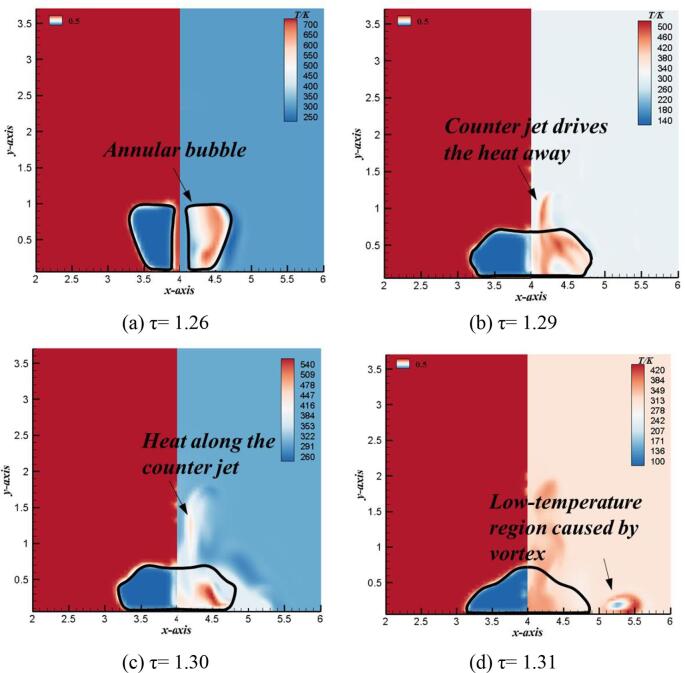
Transient evolutions of numerical bubble shapes (left) and temperature distributions (right) during bubble rebound stage (γ = 1.2).

Fig. 10 shows the comparison of numerical temperature and bubble shapes in the rebound stage. Compared with the collapse stage, due to the gradual expansion of the bubble volume in the rebound stage, the compressible vapor with high temperature inside the bubble pushes the liquid and does negative works on it, resulting in the rapid decrease of the temperature in the bubble. However, due to the phenomenon of counter jet produced in the rebound stage of bubble near the rigid wall, the thermodynamic effect of bubble has its local particularity in the rebound stage. As shown in Fig. 9 (a), in the rebound stage, the bubble firstly forms an annular shape and store the heat inside.

As shown in Fig. 10 (a), bubble splits into two parts, namely, the lower expandable sub-bubble and the upper counter jet. Among them, the sub-bubble expands continuously in the rebound stage, does negative works on the liquid outside the bubble and reduces the temperature of the vapor inside the bubble. However, the counter jet separates the part of fluid medium with high-temperature from the interior of the bubble and drives it away from the rigid wall. Therefore, the high temperature gas appears in the center of the bubble, and the temperature is lower near the rigid wall. As shown in Fig. 10 (c), with the increase of bubble volume, the temperature in the center of the bubble decreases rapidly, while the high-temperature vapor gradually distributes around the bubble. At the same time, there is a high temperature region in the counter jet. Fig. 10 (d) show the temperature distribution when a counter jet is generated at the top surface of the bubble. The temperature in the middle of the bubble rapidly decreased to about 350 K, and the temperature of the high temperature gas near the rigid wall also rapidly decreased to below room temperature. It can be seen that the vortex structure is an important energy dissipation mechanism, resulting in the local low temperature area around the bubble near the surface of the rigid wall [26]. This is because the pressure and velocity at the center of the vortex structure will decrease at the same time. According to the law of energy conservation, it can be seen that with constant potential energy, the static pressure energy and kinetic energy of the fluid decrease at the same time, so the internal energy consumption of the vortex structure increases. In addition, the counter jet takes a lot of high temperature vapor away from the bubble and moves away from the rigid wall. This avoids the temperature drop of the high-temperature vapor in the counter jet caused by the external work of the lower expanding sub-bubble and the dissipation effect of the vortex structure.

**Fig. 11:**
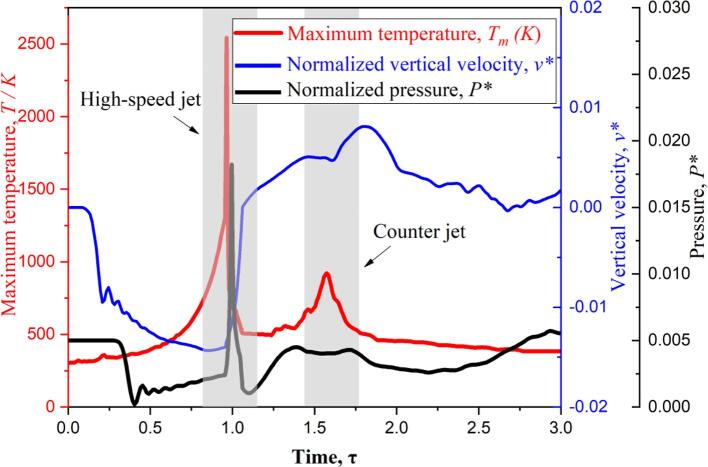
Comparison of temperature, normalized vertical component of velocity, and normalized pressure at the bubble center (γ = 1.2).

In order to further quantitatively study the relationship between the flow structure and the thermodynamic characteristics, Fig. 11 shows the vertical component of the dimensionless velocity, the dimensionless pressure and the transient evolution process of the temperature of the bubble at different times. The dimensionless velocity and pressure of the flow field are defined as

(21) $$v^{*}=v/c,P^{*}=P/\rho_{l}c^{2}$$

where ***v*** is the vertical component of velocity. The monitoring point is located at the center of the bubble. The two gray areas marked in the figure correspond to the two stages of high-speed jet and counter jet, respectively. The solid black, red, and blue lines represent the dimensionless vertical components of pressure, temperature, and velocity, respectively. For the shrink stage of single bubble, the vertical component of velocity increases significantly, the pressure decreases sharply, and the temperature remains stable at *T* = 300 K. For the collapse stage at τ = 1.0, the vertical component of velocity reaches v* = −0.014 and the peak value of pressure P* = 0.018 appears, which indicates the generation of the high-speed jet. At the same time, the temperature peak value (about *T* = 2517 K) of bubble also appears, showing the closely related mechanism between the high-speed jet and first temperature peak. For the rebound stage at τ = 1.5, the velocity peak shows the positive value, v* = − 0.009, which indicating the counter jet away from the rigid wall. At the same time, the temperature curve presents the second value peak about *T* = 750 K, which indicating the counter jet takes the much heat away from the sub-bubble. After that, the pressure rebounds back and forth due to the interaction of the sub-bubble, the surface of the bubble and the rigid wall, and the temperature, vertical components of velocity, and the pressure fluctuations are observed.

### 3.3 Effect of standoff distance on bubble thermodynamics

**Fig. 12:**
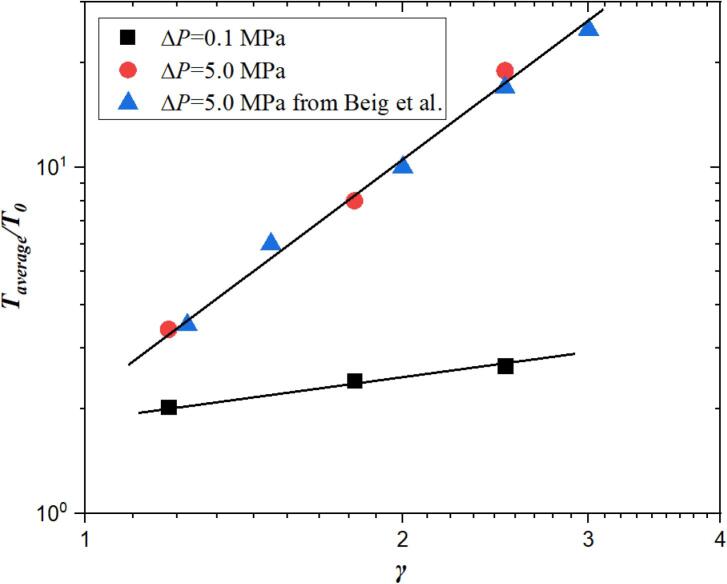
Maximum average bubble temperature versus standoff distance and pressure difference. Data is from Beig et al.[19]

As known, the standoff distance is a significantly important factor to affect the bubble thermodynamics. Therefore, Fig. 12 shows the maximum average bubble temperature versus the initial standoff distance. *T_average_* is the maximum spatially averaged temperature of the bubble achieved over the simulation for different initial stand-off distance. In order to prove the accuracy of our numerical code, the numerical results under the action of different pressure difference are compared with the works reported by Beig et al. [19]. $\Delta P\;\text{=}\;P_{l0}\;\text{-}\;P_{g0}$ is the pressure difference between initial liquid and gas. As observed, the maximum average bubble temperatures from our algorithm are consistent with that from Beig et al. [19] when ΔP= 5.0 MPa, which proves the accuracy of our numerical code. Furthermore, it is found that the maximum average bubble temperature increases with the increase of the standoff distance.

**Fig. 13:**
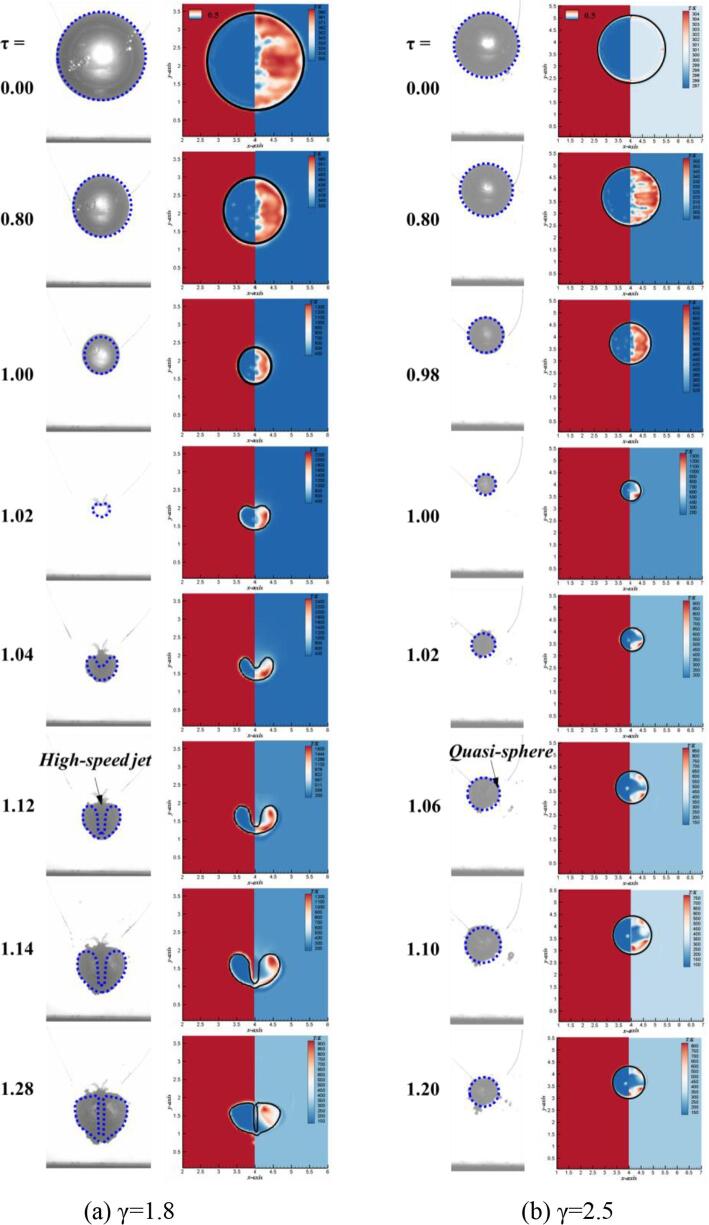
Comparisons of bubble shapes and temperature near different standoff distances. The first column is experimental bubble shapes, while the second column is numerical bubble shapes (left) and corresponding temperature contours (right).

In order to show the effect of standoff distance on the bubble thermodynamics more intuitively, in addition to the case of γ = 1.2 discussed above, Fig. 13 shows comparisons of bubble shapes and temperature near two more standoff distances, namely γ = 1.8 and γ = 2.5. The first column is experimental bubble shapes, while the second column is numerical bubble shapes and corresponding temperature contours. As shown in Fig. 13(a), the bubble with γ = 1.8 exhibits the much weaker high-speed jet during the collapsing stage when τ = 1.02–1.14, as compared with case of γ = 1.2. The high-speed jet drives the low-temperature flow outside bubble to occupy the high-temperature region inside bubble. However, compared with case of γ = 1.2, the intensity of convective heat transfer of γ = 1.8 is much weaker, due to the generation of the weaker high-speed jet. Furthermore, bubble is in the rebound stage when τ = 1.28; however, it doesn’t form the counter jet, which indicates there is no heat driven away by the counter jet. As shown in Fig. 13(b), bubble shape always presents the quasi-sphere no matter it is in collapse stage or rebound stage when γ = 2.5. The bubble neither form the high-speed jet during the collapse stage nor counter jet during the rebound stage. That’s because the Bjerknes force acting on the secondary expansion bubble is small, so the bubble is still expanding as a sphere like one. Therefore, the heat dissipation effect caused by high-speed jet and counter jet on bubble temperature is very little.

**Fig. 14:**
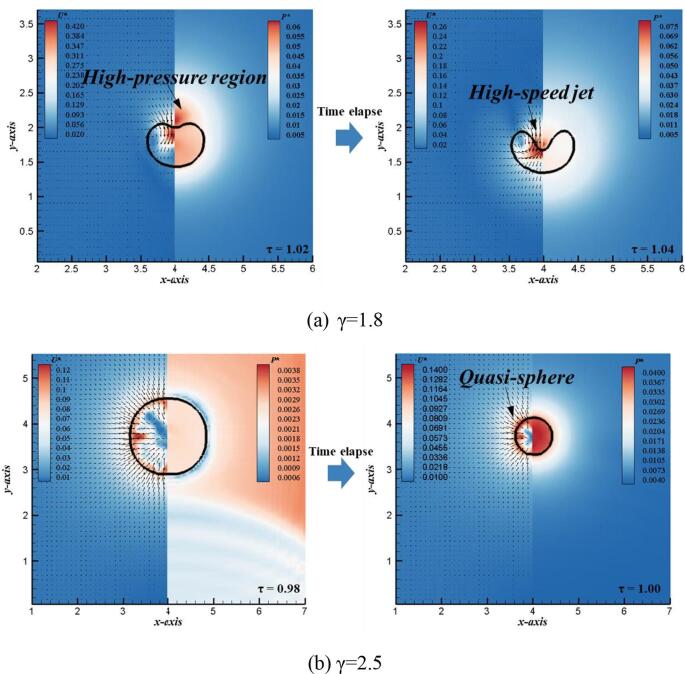
Numerical velocity (left) and pressure (right) distributions of bubble for different standoff distances to illustrate (a) the formation of high-speed jet (γ = 1.8) and (b) the formation of quasi-sphere (γ = 2.5).

In order to further investigate the different generation mechanism of the bubble thermodynamics, Fig. 14 shows the numerical results of flow structure for different initial standoffs. When the initial standoff distnace is γ = 1.8, the generation of high-speed jet is caused by the occurrence of high-pressure region at the top of bubble margin, which is same with the case of γ = 1.2. High-speed jet in case of γ = 1.8 also perform the important mechanisms to achieve energy transfer just like that of γ = 1.2. However, compared with that of γ = 1.2, the jet in case of γ = 1.8 is much weaker from the aspect of velocity contour. As for the rebound stage in γ = 1.8, the bubble doesn’t form the counter jet. That’s because the center of bubble is relatively far away from the rigid wall; therefore, there is no high-pressure region generated inside the bubble ring. When the initial standoff further increases to γ = 2.5, the fluid flows from all directions to the center of bubble, resulting in the quasi-sphere of bubble. That’s because the larger initial standoff keeps the bubble subjected in a smaller magnitude of Bjerknes force.

**Fig. 15:**
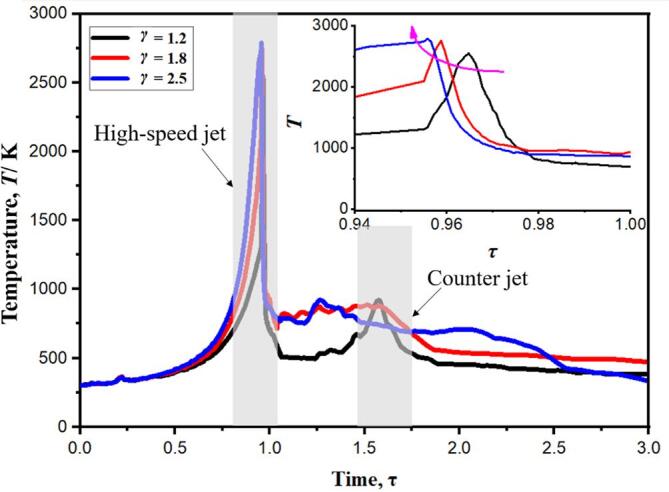
Temperature profiles at the bubble center for three different standoff distances, namely, γ = 1.2, 1.8 and 2.5 [24].

Fig. 15 shows a comparison of the internal temperature of the bubble when γ = 1.2, 1.8 and 2.5. When the time is τ = 0.95–0.97, there are obvious temperature peaks in the three conditions. The highest temperatures of γ = 1.2, 1.8 and 2.5 were 2517 K, 2762 K and 2795 K, respectively. In the collapse stage, the peak temperature T keeps increasing with the increase of the initial distance γ. Due to the increase of the initial distance γ, the Bjerknes force effect of the wall on the cavitation is significantly weakened, and the cavitation does not form an obvious high-speed jet phenomenon [31,32]. From the perspective of flow field structure, when the bubble is large in γ, it does not generate high-speed jet, so it cannot effectively achieve the heat transfer of high-temperature gas in the bubble and low-temperature liquid outside the bubble (as shown in Fig. 6). From the point of view of energy conservation, when γ is large, the work done by the wall contraction process to the compressible gas in the bubble cannot be converted into the kinetic energy of the high-speed jet, so it can be converted into the internal energy of the gas in the bubble to a large extent. When the time is τ = 1.50–1.70, the temperature peaks appear again. With the increase of γ, the temperature values of the three working conditions decrease gradually. This is because the increase of γ does not lead to the formation of reverse jet in the rebound stage, so the high temperature gas inside the cavity cannot be effectively driven away from the wall, so as to avoid the internal energy dissipation of high temperature gas by vortex structure.

## 4 Conclusions

In this paper, a fluid model considering compressibility effect is used to simulate the thermodynamic effect of bubble collapse near a rigid boundary. The model uses the improved mass conservation equation to adjust the coupling relationship between pressure and velocity in momentum equation and energy equation, and accurately captures the bubble shapes, heat transfer process, and the corresponding transient evolution process of pressure and velocity. The main conclusions are as follows:

(1) The compressible fluid model considering the effect of thermodynamics is studied and verified. The transient evolution process of bubble shapes, temperature, pressure and velocity field are accurately captured. The accuracy of the numerical model is verified by the experimental data of bubble shapes and Keller-Kolodner equation, as well as its thermodynamic equation.
(2) High-speed jet and counter jet play important roles in the process of bubble thermodynamics. In the collapse stage, the margin of the bubble shrinks rapidly and does positive works on the compressible vapor in the bubble, which produces a lot of heat. The generation of high-speed jet drives the low-temperature liquid outside the bubble to occupy the position of high-temperature vapor inside the bubble. In the rebound stage, the counter jet moving away from the wall takes a lot of heat away from the sub-bubble, avoiding the external work caused by the expansion of the sub-bubble and the temperature reduction caused by the dissipation effect of the vortex structure.
(3) The initial standoff has a significant effect on the thermodynamic effect of bubble oscillation. In the collapse stage, the temperature keeps increasing with the increase of the standoff distance. In the rebound stage, the temperature shows a downward trend with the increase of the initial position. When the initial standoff increases, the gradual disappearance of high-speed jet and counter jet is an important reason for the opposite evolution trend of temperature in collapse and rebound stage.

## CRediT authorship contribution statement

**Qidong Yu:** Resources, Funding acquisition, Supervision. **Xiaojian Ma:** Investigation, Methodology, Formal analysis, Writing - original draft. **Zhicheng Xu:** Writing - review & editing. **Jing Zhao:** Writing - review & editing. **Dapeng Wang:** Writing - review & editing. **Zhenwei Huang:** Conceptualization, Funding acquisition.

## Declaration of Competing Interest

The authors declare that they have no known competing financial interests or personal relationships that could have appeared to influence the work reported in this paper.

## Appendix A

In this part, Eqs. (A1)–(A12) are derived by Caltagirone et al.[23] and we would briefly introduce the derivation process of the updated N-S equations in order to the convenient reading for readers.

From a numerical perspective, change Eq. (A1) with a semi-discretization in time

(A1) $$\frac{\partial P}{\partial t}=\frac{\beta }{\chi_{T}}\frac{\mathrm{dT}}{\mathrm{dt}}-\frac{1}{\chi_{T}}\nabla \cdot u-u\cdot \nabla P$$

It can be expressed as

(A2) $$P^{n+1}=P^{n}-\Delta t\left(\frac{1}{\chi_{T}}\nabla \cdot u^{n+1}+u^{n+1}\cdot \nabla P^{n}-\frac{\beta }{\chi_{T}}\frac{dT^{n+1}}{\mathrm{dt}}\right)$$

where *n* is calculated step and Δ*t* is calculated time interval.

The important variables in Eq. (A2) is defined as dimensionless number

(A3) $$P^{*}=\frac{P}{\rho V_{0}^{2}},u^{*}=\frac{u}{V_{0}},t^{*}=\frac{tV_{0}}{H},T^{*}=\frac{T}{\delta T}$$

where *V_0_* is the reference speed, *H* is the characteristic length, andδTis the reference temperature. Therefore, Eq. (A3) can be expressed as

(A4) $$\frac{\partial P\text{*}}{\partial t\text{*}}=-\frac{1}{Ma^{2}}\nabla \cdot u^{*}-u^{*}\cdot \nabla P^{*}+\frac{\beta \cdot \delta T}{M{\text{a}}^{2}}\frac{dT^{*}}{dt^{*}}$$

where the sound velocity is $c=\sqrt{1/\rho \chi_{T}}$and the Mach number is $M\text{a}=\frac{V_{0}}{c}$. By introducing the definition of Gay-Lussac number Ga=β·δT, it can quantify the density differences of the working fluid under the temperature field. And the Eq. (A4) can be expressed in a non-dimensional expression as follows

(A5) $$\frac{\partial P\text{*}}{\partial t\text{*}}=-\frac{1}{Ma^{2}}\nabla \cdot u^{*}-u^{*}\cdot \nabla P^{*}+\frac{\mathrm{Ga}}{Ma^{2}}\frac{dT^{*}}{dt^{*}}$$

In the dimension analysis, the time scale of the pressure fluctuations is taken to be $\frac{H}{V_{0}}$. In order to capture the pressure waves, a time scale defined as $\frac{H}{c}$ seems more appropriate. The dimensionless set of parameters is written again as:

(A6) $$P^{*}=\frac{P}{\rho V_{0}^{2}},u^{*}=\frac{u}{V_{0}},t^{*}=\frac{\mathrm{tc}}{H},T^{*}=\frac{T}{\delta T}$$

and the non-dimensional equation for pressure becomes

(A7) $$\frac{\partial P\text{*}}{\partial t\text{*}}=-\frac{1}{\mathrm{Ma}}\nabla \cdot u^{*}-M\text{a}\cdot u^{*}\cdot \nabla P^{*}+\frac{G\text{a}}{M{\text{a}}^{2}}\frac{dT^{*}}{dt^{*}}$$

Noticing that $\delta T/T\approx \delta P/P\approx Ma^{2}$ for weakly compressible flows, the last term of Eq. (A7) is of the first order *O* (1). This can be shown by considering the following equations:

(A8) $$\frac{\mathrm{dT}}{\mathrm{dt}}=a\nabla^{2}P$$

(A9) $$\frac{\partial P^{\text{*}}}{\partial t^{\text{*}}}=-\frac{1}{\mathrm{Ma}}\nabla \cdot u^{*}-M\text{a}\cdot u^{*}\cdot \nabla P^{*}+\frac{1}{M\text{a}}\frac{1}{P\text{e}}Ga\nabla^{2}T^{\text{*}}$$

where *a* is the thermal diffusivity and $\mathrm{Pe}=\frac{HV_{0}}{a}$is the Peclet number. For low Mach number and highly inertial flows, the relations (A9) are generally satisfied:

(A10) Ma→0,Ga<<1,Pe>>1

Thus, the two last terms in Eq. (A9) can be neglected in Mach numbers limited to zero, including highly compressible flows, involving fluids with a high compressibility coefficient. The resulting equation for pressure is:

(A11) $$\frac{\partial P^{*}}{\partial t^{*}}=-\frac{1}{\mathrm{Ma}}\nabla \cdot u^{*}$$

Change the above formula into the actual variable as

(A12) $$\frac{\partial P}{\partial t}=-\frac{1}{\chi_{T}}\nabla \cdot u$$

where the gas is *χ_T air_* = 9.869 × 10^-6^ Pa^−1^, and the liquid is *χ_T water_* = 0.444 × 10^-9^ Pa^−1^.

## Appendix B

In order to prove the relationship of $\beta T{\left(\frac{\partial P}{\partial T}\right)}_{\rho }\;\text{=}\;\rho \left(c_{p}-c_{v}\right)$, three important equations must be firstly established, namely, the first d*s* equation, the second d*s* equation, and the general relation equation of (*c_p_-c_v_*). Then, the detailed derivation process is shown as follows:

**(1) The first d*s* equation.** If *T* and *ν* are set as independent variables of specific entropy, namelys=s(T,v)

(B1) $$\text{d}s={\left(\frac{\partial s}{\partial T}\right)}_{v}dT+{\left(\frac{\partial s}{\partial v}\right)}_{T}dv$$

where *s* is specific entropy. According to Maxwell equation,

(B2) $${\left(\frac{\partial s}{\partial v}\right)}_{T}\;\text{=}\;{\left(\frac{\partial P}{\partial T}\right)}_{v}$$

According to the chain relation and the definition of specific heat capacity

(B3) $${\left(\frac{\partial s}{\partial T}\right)}_{v}{\left(\frac{\partial T}{\partial u}\right)}_{v}{\left(\frac{\partial u}{\partial s}\right)}_{v}=1$$

where *u* is internal energy.

(B4) $${\left(\frac{\partial s}{\partial T}\right)}_{v}\;\text{=}\;\frac{{\left(\frac{\partial u}{\partial T}\right)}_{v}}{{\left(\frac{\partial u}{\partial s}\right)}_{v}}=\frac{c_{V}}{T}$$

Then we can obtain

(B5) $$\text{d}s=\frac{c_{V}}{T}dT+{\left(\frac{\partial P}{\partial T}\right)}_{v}dv$$

Therefore, Eq (B5) is called as the first d*s* equation.

**(2) The second d*s* equation**. If *T* and *P* are set as independent variables of specific entropy, namelys=s(T,P)

(B6) $$\text{d}s={\left(\frac{\partial s}{\partial T}\right)}_{P}dT+{\left(\frac{\partial s}{\partial P}\right)}_{T}dP$$

Due to the equations as following

(B7) $${\left(\frac{\partial s}{\partial P}\right)}_{T}\;\text{= -}\;{\left(\frac{\partial P}{\partial T}\right)}_{P},{\left(\frac{\partial s}{\partial T}\right)}_{P}\;\text{=}\;\frac{{\left(\frac{\partial h}{\partial T}\right)}_{P}}{{\left(\frac{\partial h}{\partial s}\right)}_{P}}=\frac{c_{P}}{T}$$

where *h* is specific enthalpy. Then

(B8) $$\text{d}s=\frac{c_{P}}{T}dT+{\left(\frac{\partial v}{\partial T}\right)}_{P}dP$$

Therefore, Eq (B8) is called as the second d*s* equation.

**(3) The general relation equation of (*c_p_-c_v_*).** By combining the first d*s* equation (B5) and the second d*s* equation (B8), we can obtain

(B9) $$c_{P}dT\;\text{-}\;T{\left(\frac{\partial v}{\partial T}\right)}_{P}dP\;\text{=}\;c_{v}dT\;\text{+}\;T{\left(\frac{\partial P}{\partial T}\right)}_{v}dv$$

Then

(B10) $$\text{d}T=\frac{T{\left(\frac{\partial v}{\partial T}\right)}_{P}}{c_{P}-c_{v}}dP+\frac{T{\left(\frac{\partial P}{\partial T}\right)}_{v}}{c_{P}-c_{v}}dv$$

When $T\;\text{=}\;T\left(v,P\right)$, we can obtain

(B11) $$\text{d}T={\left(\frac{\partial T}{\partial v}\right)}_{P}dv+{\left(\frac{\partial T}{\partial P}\right)}_{v}dP$$

By contrasting Eq. (B10) and Eq. (B11), we can obtain

(B12) $${\left(\frac{\partial T}{\partial v}\right)}_{P}\;\text{=}\;\frac{T{\left(\frac{\partial P}{\partial T}\right)}_{v}}{c_{p}-c_{v}},{\left(\frac{\partial T}{\partial P}\right)}_{v}\;\text{=}\;\frac{T{\left(\frac{\partial v}{\partial T}\right)}_{P}}{c_{p}-c_{v}}$$

Therefore, the equation above can be transformed as

(B13) $$c_{p}-c_{v}\;\text{=}\;T{\left(\frac{\partial v}{\partial T}\right)}_{P}{\left(\frac{\partial P}{\partial T}\right)}_{v}$$

Eq. (B13) is called as the general relation equation of (*c_p_-c_v_*).

**(4) Final equation.** By substituting coefficient of thermal expansion$\beta \;\text{=}\;\frac{1}{v}{\left(\frac{\partial v}{\partial T}\right)}_{P}$ in to Eq (B13), we can obtain

(B14) $$c_{p}-c_{v}\;\text{=}\;v\beta T{\left(\frac{\partial P}{\partial T}\right)}_{v}$$

and then

(B15) $$\beta T{\left(\frac{\partial P}{\partial T}\right)}_{v}\;\text{=}\;\frac{c_{p}-c_{v}}{v}$$

By substituting $\rho \;\text{=}\;\frac{1}{v}$ in to Eq (B15), we can finally obtain

(B16) $$\beta T{\left(\frac{\partial P}{\partial T}\right)}_{\rho }\;\text{=}\;\rho \left(c_{p}-c_{v}\right)$$

## References

[1] Aktas B., Atlar M., Turkmen S., Shi W., Sampson R., Korkut E., Fitzsimmons P. (2016). Propeller cavitation noise investigations of a research vessel using medium size cavitation tunnel tests and full-scale trials. Ocean Eng..

[2] Coutier-Delgosha O. (2019). Special issue on aerospace and naval propulsion [J]. J. Fluids Eng..

[3] Wu P., Bai L., Lin W., Wang X. (2018). Mechanism and dynamics of hydrodynamic-acoustic cavitation (HAC). Ultrason. Sonochem..

[4] Thiemann A., Holsteyns F., Cairós C., Mettin R. (2017). Sonoluminescence and dynamics of cavitation bubble populations in sulfuric acid. Ultrason. Sonochem..

[5] Ma X., Huang B., Zhao X., Wang Y., Chang Q., Qiu S., Fu X., Wang G. (2018). Comparisons of spark-charge bubble dynamics near the elastic and rigid boundaries. Ultrason. Sonochem..

[6] Ma J., Hsiao C.-T., Chahine G.L. (2015). Modelling Cavitating Flows using an Eulerian-Lagrangian Approach and a Nucleation Model. J. Phys.: Conf. Ser..

[7] Brujan E.A., Nahen K., Schmidt P. (2001). Dynamics of laser-induced cavitation bubbles near an elastic boundary. J. Fluid Mech..

[8] Dular M., Coutier-Delgosha O. (2013). Thermodynamic effects during growth and collapse of a single cavitation bubble. J. Fluid Mech..

[9] Flannigan D.J., Suslick K.S. (2005). Plasma formation and temperature measurement during single-bubble cavitation. Nature.

[10] FLINT E.B., SUSLICK K.S. (1991). The Temperature of Cavitation. Science.

[11] Liu X., Long Z., He J., Liu X., Hou Y., Lu J., Ni X. (2013). Temperature effect on the impact of a liquid-jet against a rigid boundary. Optik.

[12] Brennen C.E. (1995). Cavitation and Bubble Dynamics.

[13] Alhelfi A., Sunden B. (2015). Simulations of cryogenic cavitation of low temperature fluids with thermodynamics effects. Energy.

[14] Ma X., Wang C., Huang B., Wang G. (2019). Application of two-branch deep neural network to predict bubble migration near elastic boundaries. Phys. Fluids.

[15] Zhang A.M., Li S., Cui J. (2015). Study on splitting of a toroidal bubble near a rigid boundary. Phys. Fluids.

[16] Zhang A., Sun P., Ming F. (2015). An SPH modeling of bubble rising and coalescing in three dimensions. Comput. Methods Appl. Mech. Eng..

[17] Yang Y.u., Shan M., Kan X., Shangguan Y., Han Q. (2020). Thermodynamic of collapsing cavitation bubble investigated by pseudopotential and thermal MRT-LBM. Ultrason. Sonochem..

[18] Cheng N., Guo Y., Peng C. (2019). A simulation of bubble growth on heating surface in subcooled boiling water based on the heat flows derived by experiment. Int. J. Heat Mass Transf..

[19] Beig S.A., Aboulhasanzadeh B., Johnsen E. (2018). Temperatures produced by inertially collapsing bubbles near rigid surfaces. J. Fluid Mech..

[20] Kyriazis N., Koukouvinis P., Gavaises M. (2017). Numerical investigation of bubble dynamics using tabulated data. Int. J. Multiph. Flow.

[21] Qin Z., Alehossein H. (2016). Heat transfer during cavitation bubble collapse. Appl. Therm. Eng..

[22] Christian C.M. (2012). Modeling Laser-generated Cavitation Bubbles[M]. Pennsylvania State University.

[23] Caltagirone J.P., Vincent S., Caruyer C. (2011). A multiphase compressible model for the simulation of multiphase flows. Comput. Fluids.

[24] Ma X., Zhao X., Huang B. (2019). Physical investigation of non-spherical bubble collapse near a rigid boundary. J. Hydrodynamics.

[25] Vincent S., Balmigere G., Caruyer C. (2009). Contribution to the modeling of the interaction between a plasma flow and a liquid jet [J]. Surf. Coat. Technol..

[26] Zhang M., Chang Q., Ma X. (2019). Physical investigation of the counterjet dynamics during the bubble rebound[J]. Ultrason. Sonochem..

[27] Hirt C.W., Nichols B.D. (1981). Volume of fluid (VOF) method for the dynamics of free boundaries. J. Comput. Phys..

[28] Hung C.F., Hwangfu J.J. (2010). Experimental study of the behaviour of mini-charge underwater explosion bubbles near different boundaries. J. Fluid Mech..

[29] Nichols B., Hirt C., Hotchkiss R. (1980). SOLA-VOF: A solution algorithm for transient fluid flow with multiple free boundaries.

[30] Huang G., Zhang M., Ma X. (2020). Dynamic behavior of a single bubble between the free surface and rigid wall[J]. Ultrason. Sonochem..

